# Patient characteristics and palliative care eligibility in public vs. private emergency care: A cross-sectional observational study

**DOI:** 10.1016/j.clinsp.2025.100859

**Published:** 2026-02-06

**Authors:** Carla Bertelli, Cristina Terzi, Marileise Roberta Fonseca, Felipe Cecílio, Luciana Nucci, Luana Aranha, Paulo de Campos, Fernanda Engelbrecht, Renan Oliveira, Elisa Teixeira Mendes

**Affiliations:** a Pontifícia Universidade Católica de Campinas, Faculdade de Ciências Biológicas, Campinas, SP, Brazil; b Department of Palliative Medicine, Universidade Estadual de Campinas, Campinas, SP, Brazil; c Vera Cruz Hospital, Campinas, SP, Brazil; d São Luiz Hospital, Campinas, SP, Brazil

**Keywords:** Palliative care, Emergency department, Access, Health care system

## Abstract

**Context:**

The rapid aging of the Brazilian population in recent decades has strained the healthcare system. A lack of chronic patient absorption in primary care has led to overloaded emergency departments, which handle preventable decompensation and care that could be managed at home.

**Objectives:**

To identify the demand for patients eligible for Palliative Care (PC), among patients in four Emergency Departments (ED) in a metropolitan region in Brazil.

**Methods:**

This observational, cross-sectional study involved four emergency departments: two public university hospitals and two private hospitals. Patient data on gender, age, visit reasons, and comorbidities were collected, along with the Supportive and Palliative Care Indicators Tool (SPCIT-BR™), Palliative Performance Scale (PPS) assessments, and opioid use information.

**Results:**

Of the 270 patients, 101 (37.4 %) were eligible for PC. Of these eligible patients, the median age was 70-years, 54.5 % were male and public EDs had 9-fold higher odds of palliative care eligibility. Age, dyspnea, and fever predicted palliative care eligibility.

**Conclusions:**

The present findings demonstrate the necessity of further implementation of palliative care programs, particularly in the public health system. Such integration could lead to a decrease in unnecessary ED visits and home-based care that reflects patient wishes.

## Highlights

• 37.4 % of emergency patients were eligible for palliative care.
• Public EDs had 9-fold higher odds of palliative care eligibility.
• Pain was the most reported symptom among eligible patients.
• Opioid use was low despite high symptom burden in PC patients.
• SPICT-BR™ and PPS tools revealed unmet needs in emergency care.

## Key message

As Brazil’s population ages, the healthcare system faces rising pressure and excessive demand for primary care. This study found that 37.4 % of 270 patients in four emergency departments were eligible for palliative care, primarily in public hospitals, highlighting the need to improve access to Palliative Care in primary care.

In 2018, the World Health Organization (WHO) defined PC as an approach that enhances the Quality of Life (QOL) of patients (both adults and children) and their families who are living with life-threatening diseases. Hence, the definition is expanded to an attitude that avoids and attenuates suffering through the early recognition, evaluation, and treatment of pain as well as of other physical, psychosocial, and spiritual issues.^1^ One of the most important criteria for quality of death is the quality of palliative care ‒ managing pain, providing comfort, and attending to the well-being of the patient and patient's family, says it all and should be analyzed further. In 2015, The Economist Quality of Death Index for Brazil reached 42nd, showing that Brazil is on the list of countries with the worst indicators for end-of-life care.^2^ The first place on this list emphasizes the need to overhaul educational policies for the health care professionals, palliative care protocol, and research and development in this area.^3^^,^^4^

The Brazilian population has rapidly aged in recent decades, significantly impacting the healthcare system, which has struggled to adapt to this change. In Brazil, the demand for Palliative Care (PC) exceeds the available supply, primarily concentrated in hospitals (81.1 %)^5^ leading to inadequate primary care absorption of chronic patients. As a result, EDs are overwhelmed with complications that could be prevented and care that could be handled at home. The time-pressured atmosphere working in the ED does not allow proper contact between health workers with patients, and there is no possibility of creating a kind of closer and more humanized relationship^6^.

Persistent structural and cultural obstacles impede early incorporation of palliative care in health systems, and bias towards hospital-based interventions rather than integrated, patient-centred models in the community involving primary care.^7^^,^^8^

In Brazil, there is a lack of robust data on palliative care patients, particularly studies that evaluate these demands in the ED. Considering this scenario, the present study aimed to estimate the proportion and characteristics of patients eligible for palliative care who visited public and private ED during the investigation period. Identifying these factors will help better understand and organize the healthcare system in the country for patients requiring palliative care. This study aimed to compare patients eligible for palliative care by analyzing the distribution of PPS,^9^ characteristics of life-threatening diseases, and symptoms in public and private emergency departments in a Brazilian city.

## Materials and methods

### Study design

This was a cross-sectional observational study, designed to assess the prevalence and clinical characteristics of patients eligible for Palliative Care (PC) in emergency settings. The study design is stated here, in accordance with STROBE recommendations.

There was no formal sample size calculation as the current study used a census design involving all adult patients seen at four participating EDs over 24 h. A post-hoc power analysis revealed that the full sample (*n* = 270; 101 PC-eligible) had > 80 % power to detect an odds ratio ≥ 2.0 for the relationship between ED type (public vs. private) and PC eligibility (α = 0.05).

### Ethical aspects

This research was approved by the Ethics Committee and the Institutional Review Board (IRB) of the Pontifical Catholic University of Campinas (Protocol: 5.898.910). Written informed consent was obtained from all patients or their legal guardians before participation. Those patients not wishing to participate were excluded.

### Setting

The study was conducted in four emergency departments in Campinas, a city located 100 km outside the city of São Paulo, with a population of approximately 1100,000. Two are University teaching hospitals where public healthcare is provided, and in two other private healthcare hospitals, with an average of 20 thousand emergency visits per month. Data collection occurred during 2 days (August 21 and 22, 2022) in 12-hour shifts at each ED for 24 h at each institution.

### Participants and eligibility criteria

The eligible participants were patients over 18-years of age, from spontaneous demand, or those hospitalized in the emergency room of the four participating hospitals. Excluded patients who were alone without the capacity to answer or did not agree to participate in the research.

### Data collection

All researchers were healthcare workers with basic knowledge of the PC area. They all had preparatory training with the main researcher for verification of eligibility criteria, conducting an interview using a questionnaire, and application of the SPCIT-BR™ and PPS tools. This training included rigorous standardization for using the SPICT-BR™ tool, with online meetings and consensus discussions to calibrate the interpretation of eligibility criteria, although formal inter-rater reliability was not calculated.

The questionnaire for the interview was developed specifically for this study. The interview was divided into 4 parts.

- 1st Part: epidemiological data of the patient.
- 2nd Part: questions related to the reason for visiting the emergency room, main comorbidities.
- 3rdPart: This phase involved applying the SPCIT-BR™,^10^ a clinical tool designed to identify patients in primary care and hospital settings, classifying them as either “eligible” or “ineligible” for palliative care. Additionally, the PPS, a validated and reliable tool, was used to assess the patient's functional status.
- 4th Part: evaluation of medications in use, recurrent symptoms, recurrent hospitalizations, evaluation of treatment sites.

**Fig. 1:**
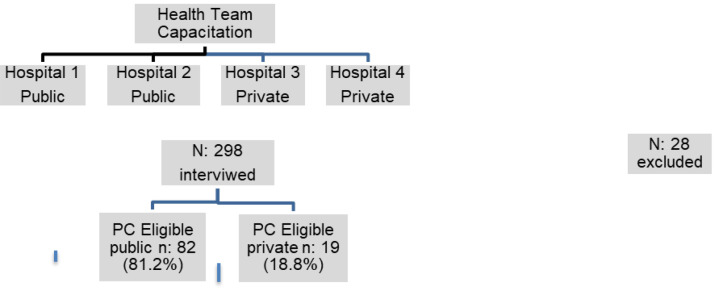
Flowchart to illustrate the recruitment process.

The researchers interviewed all patients who were present in the ED on the days selected for data collection and agreed to participate. This was done regardless of the patients' underlying disease or the reason for their visit. Fig. 1 shows the recruitment process flowchart.

### Statistical analysis

The data collection was for convenience sampling.

A comparison was made between patients eligible and not eligible for palliative care regarding their clinical, social, and epidemiological characteristics (public or private department, type of underlying disease, functionality, among other variables). Categorical variables were compared using the Chi-Square and Fisher's exact tests, as appropriate. Continuous variables were described as the median and interquartile range (Q1‒Q3), after being tested for normality using the Shapiro-Wilk test, and were then compared using the Mann-Whitney test, as appropriate. Multivariable logistic regression was used to investigate eligibility for PC, as well as the associations between the studied variables and the type of health care (public or private). The statistical significance level was set to 5 % (*p* < 0.05). The data obtained were exported to Microsoft® Office Excel and later analyzed using SAS Studio v3.82 software.

### Reporting guidelines

The manuscript was developed and reviewed using the STROBE checklist to ensure transparent, complete, and standardized reporting of observational research. All relevant items from the checklist were addressed throughout the manuscript.

## Results

**Fig. 2:**
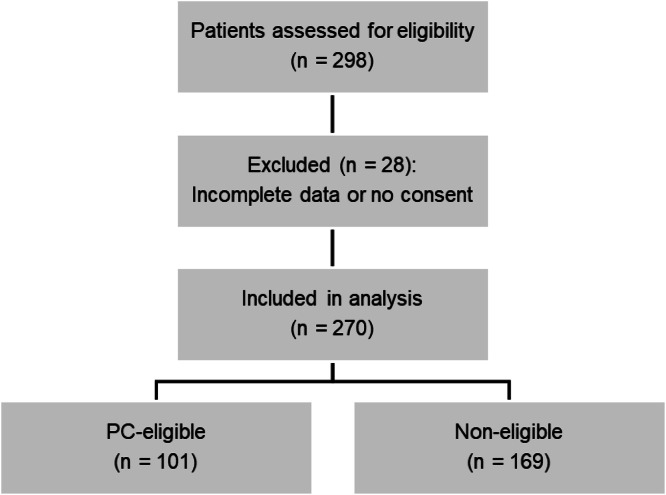
Flowchart of patient inclusion.

A total of 298 patients were initially interviewed across the four emergency departments. However, 28 patients were excluded due to incomplete data or refusal to sign the informed consent, resulting in a final sample of 270 participants included in the analysis (Fig. 2).

After applying the SPICT-BR™ eligibility tool, the authors obtained a total of 101 (37.4 %) patients with an indication for palliative care.

Analyzing the distribution of patients in general, median (Q1‒Q3) age was 58 (42‒72) years, most of them were female (54.4 %), came from public departments (57.0 %), and the most reported symptom in the emergency room was pain (37.1 %). There were statistically significant differences in age, gender, health department, and symptoms such as pain, dyspnea, fever and flu-like syndrome between patients eligible and not eligible for PC (Table 1).

**Table 1:** Epidemiological characteristics and symptoms of all patients interviewed and those eligible for palliative care. Distribution of the interviewed population according to the profile of the health department, whether public or private.

Variable	Total patients ( %)	Patients not eligible for PC ( %)	Patients eligible for PC ( %)	p-value^a^
Number of Patients	270 (100)	169	101	
Sex ( %)				
Male	123 (45.5)	68 (40.3)	55 (54.5)	0.0232
Female	147 (54.5)	101 (59.7)	46 (45.5)	
Age (years)				
Average (+)	57.7 ± 18.7	51.3 ± 18.2	68.4 ± 14.2	
Public Department	154 (57)	72 (42.6)	82 (53)	<0.0001
Private Department	116 (43)	97 (57.4)	19 (16)	
Symptoms ( %)				
Pain	100 (37.1)	71 (41.5)	29 (28.2)	0.0360
Dyspnea	35 (12.9)	8 (4.8)	27 (26.3)	<0.0001
Neurological^b^	35 (12.9)	22 (13.0)	13 (12.6)	0.9723
Fatigue and inappetence	15 (5.1)	6 (3.5)	9 (8.7)	0.0628
Anxiety	5 (1.8)	5 (3.0)	0	0.1607
Fever	10 (3.7)	3 (2.0)	7 (6.8)	0.0434
Flu-like syndrome	22 (8.1)	21 (12.3)	1 (0.9)	<0.0001
Gastrointestinal	16 (5.5)	13 (7.6)	3 (2.9)	0.1118
Other	35 (12.9)	21 (12.3)	14 (13.6)	0.7340
Associations of two or more symptoms	5 (1.8)	3 (2.0)	2 (1.9)	

PC, Palliative Care.

a Chi-Square test.

b Delirium, aphasia, motor changes, loss of consciousness, dizziness or vertigo.

On multivariable logistic regression (Table 2), older age (OR = 1.08; 95 % CI 1.05–1.10), public emergency department attendance (OR = 9.19; 95 % CI 4.06–20.80), dyspnea (OR = 5.24; 95 % CI 1.89–14.51) and fever (OR = 10.64; 95 % CI 1.92–58.89) were independently associated with increased likelihood of palliative care eligibility. A statistically nonsignificant association was also observed between the sex of the patients to flu-like symptoms.

**Table 2:** Logistic regression analysis for Palliative Care (PC) eligibility of the interviewed population.

Characteristics	Crude (95 % CI) OR	p-value	Adjusted (95 % CI) OR	p-value
Age (years)	1.06 (1.04‒1.08)	<0.001	1.08 (1.05‒1.10)	<0.001
Sex				
Male	1.78 (1.08‒2.92)	0.024	1.31 (0.68‒2.52)	0.427
Female	Ref.		Ref.	
Health Department				
Public	5.81 (3.24‒10.43)	<0.001	9.19 (4.06‒20.80)	<0.001
Private	Ref.		Ref.	
Symptoms				
Pain	0.57 (0.34‒0.97)	0.037	0.59 (0.29‒1.20)	0.145
Dyspnea	7.34 (3.18‒16.93)	<0.001	5.24 (1.89‒14.51)	0.002
Fever	4.12 (1.04‒16.31)	0.044	10.64 (1.92‒58.89)	0.007
Flu-like syndrome	0.07 (0.01‒0.50)	0.009	0.14 (0.01‒1.52)	0.106

CI, Confidence Interval; OR, Odds Ratio; PPS, Palliative Performance Scale; Ref., Reference category.

Among patients eligible for palliative care, the authors observed low use of morphine, with only 10.9 % using opioids to control symptoms. Among life-threatening diseases, malignant neoplasms are in the first place, the most prevalent in PC patients. In the public healthcare department, malignant neoplasm remained the most prevalent disease, followed by cardiopathy, Chronic Kidney Disease (CKD), and Chronic Obstructive Pulmonary Disease (COPD). In private hospitals, there was a predominance of diseases related to elderly patients, such as dementia and frailty syndrome. Dementia was more prevalent in the private sector, and this difference was statistically significant (Table 3).

**Table 3:** Description of life-threatening diseases and PPS in the population eligible for palliative care and comparison between department profiles.

Characteristics	Total of patients	Department		
		Public	Private	p*-*value
	n ( %)	n ( %)	n ( %)	
Number of patients	101 (100.0)	82 (81.2)	19 (18.8)	
Underlying disease ( %)				
Malignant neoplasm	35 (34.7)	30 (36.6)	5 (26.3)	0.3967^a^
Cardiopathy	25 (24.8)	23 (28.0)	2 (10.5)	0.1453^b^
Dementia/ Frailty syndrome of the elderly	17 (16.8)	7 (8.5)	10 (52.6)	<0.0001^b^
Chronic Obstructive Pulmonary Disease	12 (11.9)	9 (11.0)	3 (15.8)	0.6931^b^
Chronic kidney disease	10 (9.9)	10 (12.2)	0 (0.0)	0.2012^b^
Hepatopathy	6 (5.9)	6 (7.3)	0 (0.0)	0.5909^b^
Neurological	6 (5.9)	6 (7.3)	0 (0.0)	0.5909^b^
Other	3 (3.0)	2 (2.4)	1 (5.3)	0.4686^b^
Associations of 2 or more diseases	13 (12.9)	11 (13.4)	2 (10.5)	1.0000^b^
PPS				
Median (Q1‒Q3)	50 (40–70)	55 (50–70)	50 (30–60)	0.024^c^
≤30	19 (18.8)	12 (14.6)	7 (36.8)	0.042^a^
40–60	55 (54.5)	45 (54.9)	10 (52.6)	
≥70	27 (26.7)	25 (30.5)	2 (10.5)	

PPS, Palliative Performance Scale; Q1, First Quartile; Q3, Third Quartile; PPS ≤ 30, End-of-life stage; PPS 40–60, Moderate functional impairment, high symptom burden; PPS ≥ 70, Preserved function or early decline.

a Chi-square test.

b Fisher’s Exact test.

c Mann-Whitney test.

The median (Q1‒Q3) PPS was 50 (40‒70), and in the public department, it was higher than in the private department, with median values of 55 and 50, respectively. When the authors segmented the PPS into three ranges, according to the score (PPS ≤30, 40‒60, ≥70), the authors observed PPS between 40‒60 in 54.5 % of the eligible patients. This range of PPS was also prevalent in the population with indications for palliative care in the public and private departments: 54.9 % and 52.6 % (Table 3).

Within the group of eligible patients, increasing age was associated with a lower likelihood of receiving care in the public health system compared to the private sector (OR = 0.90; 95 % CI 0.83‒0.97).

**Fig. 3:**
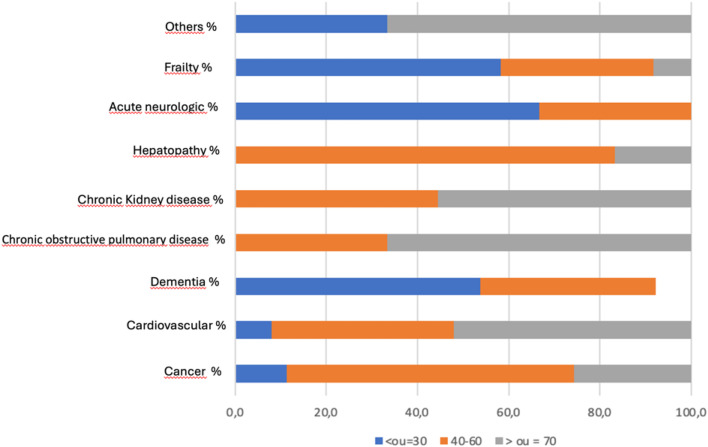
Relationship between PPS and diseases in patients eligible for PC.

In the in-depth analysis of the PPS, patients eligible for palliative care with elderly frailty syndrome, acute neurological diseases, and dementia mainly had a PPS of 30 or less. On the other hand, most patients eligible for palliative care with malignant neoplasms and liver disease had a PPS between 40 and 60. Finally, patients with CKD, COPD, and heart disease had a PPS of 70 or higher (Fig. 3).

Regarding routine follow-up for underlying diseases, most patients (55.5 %) were receiving care at an outpatient clinic, while 21.8 % had no prior follow-up.

## Discussion

Population aging, associated with the increase in chronic, degenerative, and oncological diseases, increases the demand for palliative care, especially in underdeveloped places such as Brazil. In the country, Law 8080/199,0^10^ declares health as a fundamental right and mandates the State to provide the conditions for its full realization, particularly in public departments, called Unified Healthcare System (SUS).

To the best of the authors knowledge, there are no studies in the literature that make this comparative analysis between health departments and the degree of eligibility of palliative patients, but the authors know that the demand in the public department, according to the population that depends on the Unified Healthcare System (SUS), is higher. Most palliative care services in Brazil, 159 units (67.8 %), are offered through public healthcare. Of these, 123 units (52.5 %) are exclusively administered by SUS, and 32 (15.3 %) operate in partnership with private institutions.

The fact that only 16 % of private and 53 % of public patients of the clinical population were palliative care-eligible is likely due to systemic inequalities rather than to clinical disparities. In Brazil, a limited offer of outpatient and home-based care in the public sector tends to contribute to late presentation and clinical severity. These systems' barriers ‒ chronic underfunding, disjointed care coordination, regional variation ‒ exacerbate the unmet need for palliative care in the emergent setting. Even after age and clinical presentation adjustment, patients at public EDs were significantly more likely to have palliative care eligibility, emphasizing the manner in which systemic inequities influence the potential for timely care.

The structural confounders in public/private healthcare comparisons are indeed important to consider. Although individual income and economic variables were not available in the present dataset, it's worth noting that all patients were admitted to emergency departments in the metropolitan area of Campinas, reducing geographical variations between rural and urban residency. To control for possible confounding, the authors conducted multivariable logistic regression analyses (Table 2, Table 4), adjusting for key clinical and demographic factors. Receiving care at a public emergency department remained significantly associated with an increased likelihood of palliative care eligibility (adjusted OR = 9.19; 95 % CI 4.06, 20.80) even after adjustment. The authors acknowledge (in our revised Discussion) that there could be unmeasured socioeconomic confounders that continue to influence these findings, and the authors suggest that in future research, more detailed sociodemographic information should be taken into account to further support causal inferences.

**Table 4:** Multivariable logistic regression identifying predictors of palliative care eligibility in public emergency settings.

Characteristics	Crude (95 % CI) OR	p-value	Adjusted (95 % CI) OR	p-value
Age (years)	0.87 (0.81‒0.93)	<0.001	0.90 (0.83‒0.97)	0.004
Dementia/ Frailty syndrome of the elderly	0.08 (0.03‒0.28)	<0.001	0.26 (0.05‒1.31)	0.102
PPS				
≤ 30	Ref.			
40 –60	2.62 (0.83‒8.35)	0.102	0.76 (0.14‒4.07)	0.753
≥ 70	7.29 (1.31‒40.54)	0.023	0.87 (0.09‒8.94)	0.910

CI, Confidence Interval; OR, Odds Ratio; PPS, Palliative Performance Scale; Ref., Reference category.

When considering prevalence estimates, it is crucial to account for the population of interest and interpret comparisons across studies with different methods. While study found that a notable percentage of ED patients were PC-eligible, direct comparisons with studies like the Belgian one (47 % eligibility) that focused on patients > 75-years must be made with caution due to differing age cohorts and methodologies. Therefore, the authors emphasize the need for further benchmarking studies across diverse care settings and age groups, utilizing validated tools such as SPICT™, to provide clearer and more generalizable insights into PC eligibility.

In the literature, one study in Austria^11^ showed only 13.2 %^12^ demand for PC, and a Canadian study found 26.6 %. A Belgian^13^ study that used the SPICT-BR™ tool had a higher eligibility rate (47 %) but only evaluated populations over 75-years of age.

Patients with dementia frequently use the ED –particularly those with a history of hospitalization stay, or enrollment in home health services –and may benefit from targeted interventions during or before the ED encounters to reduce avoidable utilization and ensure goal-concordant care.^14^^,^^15^

The key findings of the present study showed a high percentage of patients in the ED eligible for palliative care; these results suggest an additional cause of overcrowding in emergency departments. Neoplasia was the most prevalent disease, with pain and dyspnea being the most common symptoms, which corroborates other studies,^16^^,^^17^ although very few were using morphine.

As is commonly known, morphine is an important global marker of quality in the care of palliative patients, and the use of morphine in milligrams (mg) per capita in each country is evaluated worldwide to understand how the treatment of these patients is progressing. In the latest analysis of the Latin American Atlas of Palliative Care, Brazil had a per capita use of 7.5 mg of morphine, behind Chile (26.6 mg), Argentina (18.2 mg), Colombia (13 mg), and Costa Rica (12.7 mg) .^18^ In Brazil, the underuse of opioids in palliative care is associated with multiple barriers, including regulatory constraints, inadequate training among healthcare professionals, and limited public understanding of their role in symptom relief.^19^^,^^20^

Dyspnea (adjusted OR = 5.24, 95 % CI 1.89–14.51) and fever (adjusted OR = 10.64, 95 % CI 1.92–58.89) were independently associated with PC eligibility after adjustment for other variables, indicating their significance as clinical markers for emergency triage. Both symptoms are often treatable in the outpatient or home care settings. So that patients with advanced illness can receive more individualized and less hospital-bound care.^21^

The PPS is the most widely used functional tool in palliative care, and several studies have validated its use. The data related to this tool are variable in the studies, according to the location of the research, the age of the evaluated population, and the present comorbidities.^22^ The data presented herein show that 70 % of the patients eligible for PC had PPS ≤ 60; this result is consistent with a North American study^23^ that evaluated patients in a referral department in PC, where 78.8 % of the patients had a PPS ≤ 60. There are challenges in comparing studies due to varying factors such as age and specific diseases, and the fact that each study uses different intervals to stratify the PPS in its sample. The 40–60 interval assumes special importance as it designates a population that is not quite imminently dying, but suffers a significant effect on quality of life, and requires frequent ED admission and complex care, making them promising candidates for early palliative care referral.

Patients receiving palliative care are less likely than those not receiving palliative care to visit the ED in the last month of life.^24^ Palliative care in the ED can enhance quality of life while ensuring the appropriate allocation of resources. Improved home care services and enhanced collaboration among hospitals, primary care teams, and outpatient clinics could help reduce the need for ED admissions. Among the contributing factors are the lack of access to healthcare services and the failure to address patients' needs adequately. Additionally, healthcare professionals who do not fully understand the natural progression of diseases, as palliative care specialists do, may struggle to correlate acute events with disease trajectory, thereby hindering the development of an appropriate care plan.

Palliative care programs in healthcare systems can likely reduce the psychosocial stress on families, as well as the number of emergency calls, and enable more palliative care patients to die at home.

Discussions surrounding the implementation of tools for screening and assessing the need for PC in the ED, goals and advanced care planning, symptom management, and aggressive pain control are some of the cornerstones of palliative care.^25^ Some additional benefits of early palliative care interventions in the ED include resource management, improved satisfaction for patients and their families, improved outcomes, decreased length of stay, less use of intensive care units, and reduced costs, along with increased appropriate direct hospice consults.^26^^,^^27^ Palliative care should be a human right, and preventing suffering should be a key goal of public health systems.

Additionally, enhancing the education of the emergency department healthcare team in areas such as managing critical initial evaluations, initiating pain and symptom treatment, facilitating communication, and ensuring smooth transitions to other care settings is crucial.

The implementation of structured screening tools, such as SPICT-BR™, in ED could offer a strategy to identify patients eligible for early palliative care. Such protocols have demonstrated feasibility and clinical benefit in international settings, and could be adapted to the Brazilian public health context to reduce avoidable ED utilization and improve continuity of care.^13^^,^^21^

### Limitations to the study

This study has several limitations. First, the aetiologies of ED visits by palliative-eligible patients were incompletely defined, and thus clinical acuity versus potentially avoidable utilization was difficult to infer.

Another major limitation is the lack of any formal inter-rater reliability study. Since data collectors were trained in a standard way and had calibration sessions, those findings can be reproduced, although such agreements have never been statistically quantified.

All data were collected in four hospitals in Campinas, a relatively wealthy city, and more studies might be needed to reproduce the same findings in rural or underserved regions of Brazil.

While the study used a convenience sample of two consecutive and concordant days in all sites, the sample did include all adult patients who were eligible in that two-day time frame. So, neither sample size calculation was performed. Although this census strategy minimized weekly and seasonal variation, it has restricted possibilities for making causal inferences or assessing longitudinal outcomes.

Furthermore, several important covariates, including comorbidities, socioeconomic status, and insurance type, contained a significant amount of missingness, further limiting their ability to conduct more in-depth multivariable adjustment and sensitivity analyses.

Although a sample size calculation was not conducted a priori, a post-hoc analysis showed adequate power to the main comparisons made in this manuscript, justifying that these findings were internally consistent. Nonetheless, results need to be cautiously interpreted due to the exploratory and cross-sectional nature.

However, this study is able to present original data that is indispensable in characterizing the prevalence of PC needs in Brazilian emergencies, a comparison between public and private care being seldom made. These data could potentially be used as evidence in health policies and provide evidence-based, helpful information on developing focused programs for integrating palliative care in acute settings.

## Conclusion

Integrating palliative care into healthcare systems is crucial to improving care for eligible patients. It can reduce avoidable emergency department visits, support home-based end-of-life care ‒ especially within public health systems ‒ and better align care with patient preferences while optimizing healthcare resources. However, systemic barriers ‒ such as the chronic underfunding of Brazil’s Unified Health System (SUS) and poor coordination across care levels ‒ continue to limit equitable access to palliative care, particularly in emergency settings, and hinder its full integration nationwide.

## Data availability

Data are available from the corresponding author upon reasonable request.

## Funding

This research did not receive any specific grant from funding agencies in the public, commercial, or not-for-profit sectors.

## CRediT authorship contribution statement

**Carla Bertelli:** Conceptualization, Resources, Writing – original draft, Project administration. **Cristina Terzi:** Conceptualization, Methodology, Writing – original draft, Resources, Writing – review & editing, Visualization, Supervision, Project administration. **Marileise Roberta Fonseca:** Investigation. **Felipe Cecílio:** Investigation. **Luciana Nucci:** Software, Formal analysis. **Luana Aranha:** Investigation. **Paulo de Campos:** Investigation. **Fernanda Engelbrecht:** Investigation. **Renan Oliveira:** Investigation. **Elisa Teixeira Mendes:** Conceptualization, Methodology, Resources, Writing – original draft, Writing – review & editing, Visualization, Supervision, Project administration.

## Declaration of competing interest

The authors declare no conflicts of interest.

## Appendix Supplementary materials

Image, application 1

Image, application 2

Image, application 3
