# Supplementary material for: Patient characteristics and palliative care eligibility in public vs. private emergency care: A cross-sectional observational study
Source: Clinics (Sao Paulo). 2026 Feb 6;81:100859. doi: 10.1016/j.clinsp.2025.100859 (PMC12906195; doi:10.1016/j.clinsp.2025.100859)
Supplement: Supplementary file 1 [file mmc1.docx]

Table 1 – Epidemiological characteristics and symptoms of all patients interviewed and those eligible for palliative care. Distribution of the interviewed population according to the profile of the health department, whether public or private.

Table 2– Description of life-threatening diseases in the population eligible for palliative care.

Table 3 – Distribution of PPS in the population of patients eligible for palliative care and comparison between department profiles.

Fig. 1. Flowchart to illustrate the recruitment process.

Fig. 2.  Flowchart of the selection of the patients

Figure 3. Relationship between PPS and diseases in patients eligible for PC.
