# Supplementary material for: Patient characteristics and palliative care eligibility in public vs. private emergency care: A cross-sectional observational study
Source: Clinics (Sao Paulo). 2026 Feb 6;81:100859. doi: 10.1016/j.clinsp.2025.100859 (PMC12906195; doi:10.1016/j.clinsp.2025.100859)
Supplement: Supplementary file 3 [file mmc3.docx]

**STROBE Cheklist:** Patient Characteristics and Palliative Care Eligibility in Public vs. Private Emergency Care: A cross-sectional Observational Study

| **STROBE Item** | **Description** | **Assessment** | **Manuscript Page** | **Justification** |
| --- | --- | --- | --- | --- |
| 1 | Title and abstract | Adequate | p.1 | The title identifies the study design ('cross-sectional observational') and the abstract clearly presents objectives, methods, and findings. |
| 2 | Background and rationale | Adequate | p.2–3 | Background The structured rational is based on demographic stakeholders, health-system lacks and international benchmarks which are justified by the profound gap in the literature related to the palliative care needs in the Brazilian emergency context. |
| 3 | Objectives | Adequate | p.3 | The objective is well stated as estimating the proportion of patients appropriate for end-of-life care in emergency departments. |
| 4 | Study design | Adequate | p.4 | The design of the study is presented in the title ('cross sectional observational') and the abstract outlines purpose, methods, and results. |
| 5 | Setting | Adequate | p.4 | The four emergency services are described in detail, including context. |
| 6 | Participants | Adequate | p.4–5 | Inclusion/exclusion criteria are specified. All eligible patients during the two-day window were included. |
| 7 | Variables | Adequate | p.5 | All main variables (SPICT-BR™, PPS, symptoms, medications) are described and justified. |
| 8 | Data sources/measurement | Adequate | p.5–6 | Following reviewers’ comments, the Methods section was updated to include additional information for evaluator training and calibration, and the use of standardized screening instruments to support coherence upon data collection. |
| 9 | Bias | Adequate | p.6 and Revisor Response | The manuscript acknowledges potential sources of selection bias due to the two-day convenience sampling and cross-sectional design. By including **all eligible patients present during that period**, the study minimizes selection bias. |
| 10 | Study size | Partially adequate | p.6 and Revisor Response | No formal sample size calculation. Justified as a census over two days. |
| 11 | Quantitative variables | Adequate | p.6–7 | Variables are categorized in clinically meaningful groups (e.g., PPS levels). |
| 12 | Statistical methods | Adequate | p.6–7 | Logistic regression model was included to evaluate the association between type of emergency service (public vs. private) and palliative care eligibility |
| 13 | Participants flow | Adequate | p.7, Fig. 1 | Flow diagram reformulated per PRISMA-style suggestion; numbers reported. |
| 14 | Descriptive data | Adequate | p.7–9 | Detailed demographic and clinical characteristics, stratified by service type. |
| 15 | Outcome data | Adequate | p.8 | PC eligibility prevalence and comparisons between groups are clearly reported. |
| 16 | Other analyses | Partially adequate | p.9 | Logistic regression model was included |
| 17 | Key results | Adequate | p.10 | Main findings are synthesized and connected to study aims. |
| 18 | Limitations | Adequate | p.11 | Limitations (bias, reliability, design) are acknowledged transparently, after reviewers suggestions |
| 19 | Interpretation | Adequate | p.11–12 | Findings are interpreted in light of international evidence, and speculative content was revised per reviewer guidance to enhance scientific rigor. |
| 20 | Generalisability | Partially adequate | p.11 | Generalisability is addressed by acknowledging that the study setting—urban emergency services—may not reflect conditions in other regions or care levels. |
| 21 | Funding | Adequate | p.12 | Funding and conflicts of interest are clearly reported. |
